# Social and Poor-Law Problems

**Published:** 1908-04-25

**Authors:** 


### SOCIAL AND POOR-LAW PROBLEMS.

### TO MAKE ROOM FOR EPILEPTICS.

Mrs. Close, intent on getting new occupants for her Nova Scotian farm, has taken advantage of a proposal of the London County Council to provide for epileptics to make a suggestion which may appeal to the unthinking public. The London County Council has two proposals before it—either to arrange with some private institution for the reception of the 319 epileptic children within its bounds or to build and maintain one for itself. Either is undeniably expensive. As a measure of economy, Mrs. Close suggests that, to make room for a corresponding number of epileptics, the requisite number of healthy children should be taken out of London County Council institutions and handed over to the Children's Home Farm Association, at a cost of £16 per head per annum to the Council. The proposal is plausible, but there are fallacies in it which will make it impossible for the County Council, even if it were willing, to adopt it; but for the sake of the general public it is worth while to state them. Mrs. Close gives the London County Council credit for a far greater power over the children under its care than is the case. Does Mrs. Close really believe that the Education Committee of the County Council can go into any of its schools and pick out 319 children to be exported to Canada without the consent and approval of parents or relatives? This sort of compulsory emigration ended with the days of "transportation to Botany Bay." Secondly, the places thus vacated could not be filled by epileptic children. Mrs. Close says, "Epileptic children do not require special accommodation." But healthy children, if they are to remain sound in nerves as well as in body, require to be kept away from the often terrifying spectacle of epileptics. The normal has more claims on the community than the abnormal, and to scatter epileptics among institutions for other children would be an unjust strain on the officials and an unwholesome experience for the other children. Thirdly, she says that a reason for transferring the healthy children to Canada and leaving the epileptics to institution life at home is that the latter are "prevented by physical reasons from enjoying free country life." This statement makes one wonder what Mrs. Close knows of epilepsy. Free country life—that is, plenty of fresh air and wholesome manual work—is just what modern hygienists recommend for epileptics, and what the London County Council, whether they build an institution of their own or board the unfortunate children out in existing institutions, will try to give them. One reason why the cost of providing for these unfortunates will be, as Mrs. Close points out, very expensive, is just that for any suitable institution so much land is required.

### RELIEF IN KIND.

Where the recipients of out-door relief are of dubious character, it is sometimes considered advisable not to give them their allowance in money, and they receive instead an order for meat, bread, and groceries to be supplied by tradesmen selected by the Guardians. This giving of relief in kind is often resorted to when there are children to be provided for and the mother is suspected of being drunken in her habits. But the determined drinker is not to be outwitted in that way, and it is found that the pauper will often trade these orders for sums of money—of course of less value than the provisions. Prudent neighbours, whose sense of honour is not highly developed, make a good bargain with the pauper and get goods at half price or a little more. In voluntary charitable associations the same thing goes on, and is perhaps more rampant, because in the working of these there is very rarely such an efficacious check as the visit of a relieving officer who has a shrewd knowledge of one's habits. We have known of a woman who was receiving bread from two charities, and regularly sold one lot to a neighbour at a halfpenny below market price. In the same way clothes often go to others than those they are meant for, and it would be well if charitable workers realised that the prettier and more comfortable they are, the more likely they are to be sold or pawned. To prevent this, some societies deliberately make garments of two different patterns of material, while others stamp things with the mark of the association before giving them away. Voluntary charity, however, must take its chance of being cheated. It may take precautions, but it has no remedy, save to refuse further help to those who have once tricked it.

State charity is, however, in a different position, and can take some steps towards punishing those who abuse the help it offers. Thus the Lewisham Guardians have resolved to warn the police when they suspect that this kind of trading in relief tickets is indulged in, so that both seller and buyer might be prosecuted. The prosecution of the buyer is an important factor. Another suggestion that has been made in the interests of children is that when there is reason to believe that these are not getting the benefit they ought to do from relief given to their parents, the authorities should provide food under the recent Act for the feeding of school children, and deduct the amount from the relief given to the parents. It has not yet been found practical to carry this suggestion into effect, but the fact that it has been made, as well as the other difficulties and dangers we have mentioned, may serve to show that the relief of poverty is not such an easy and simple thing as some amiable people imagine.

